# Challenges and Advances in Bee Health and Diseases

**DOI:** 10.3390/vetsci10040253

**Published:** 2023-03-28

**Authors:** Giovanni Cilia, Antonio Nanetti

**Affiliations:** CREA Research Centre for Agriculture and Environment (CREA-AA), Via di Saliceto 80, 40128 Bologna, Italy

Understanding the health status of bees is crucial in assessing the epidemiology of pathogens that cause diseases in honey bee (*Apis mellifera*) colonies and wild bees. Additionally, host–pathogen interactions that impact bee health are part of a multifactorial framework that involves dynamically balancing an array of threats and resources that interact at multiple levels of scales.

In light of the extraordinary response garnered by the previous Special Issue *Honey Bee Health* [1], the objective of the current Special Issue, *Challenges and Advances in Bee Health and Diseases*, is to delve further into the domain of bee health. This includes managed honey bees as well as wild bees, with a focus on several facets of bee pathogens and bee diseases in a series of research articles. 

The theme in question is thoroughly examined across the 13 published articles, consisting of 11 original research pieces, 1 review article, and 1 brief report. These works collectively underscore the criticality of the matter at hand, as is evidenced by Figure 1.

El-Seedi and colleagues conducted a review of the honey bee immune system, examining its correlations with both nutrition and exposure to stressors [2].

A survey performed in the province of Santa Fe, Argentina, shed light on the primary factors impacting the productivity of managed honey bee colonies. These factors encompassed a range of variables, such as nuclei preparation, the number of combs in the brood chamber, bee queen replacement, and the disinfection of beekeeping equipment [3].

A study conducted in Mexico investigated the effect of genotype and climate on the resistance of European and Africanized honey bee colonies to parasitic and viral diseases. Although *Varroa destructor*, *Nosema ceranae*, deformed wing virus (DWV), and black queen cell virus (BQCV) were found in both genotypes, environmental factors did not have a significant effect on the parasitism or infection intensity of colonies of either genotype. Therefore, it was concluded that the primary factor influencing the resistance of honey bee colonies to DWV, BQCV, and *V. destructor* is the genotype of the colony itself, independent of climate [4].

A monitoring program was carried out on a total of 115 colonies located in the Marmara region of Turkey, revealing the significant prevalence of *V. destructor* and *Nosema.* Additionally, the viruses ABPV, DWV, Kakugo virus (KV), and Varroa destructor virus-1 (VDV1), which are typically transmitted via the trophic activity of Varroa mites, were found to be present in nearly all of the apiaries investigated, with a prevalence of approximately 100% [5].

A study was conducted in the Emilia-Romagna region of northern Italy, utilizing 31 apiaries to monitor the health status of managed honeybees. The most abundant pathogens identified were DWV, CBPV, and *N. ceranae*, which exhibited distinct seasonal patterns. Furthermore, a weak but significant correlation was observed between the abundance of DWV and *N. ceranae* and the geographical longitude of the apiaries, with higher values in locations situated close to the eastern coast [6].

Molecular analysis was performed to assess the presence of both *N. apis* and *N. ceranae* in 474 honey bee colonies across the Azores during the years 2014–2015. Subsequent spatiotemporal analysis until 2020 revealed the rapid expansion of *N. ceranae* in all islands in the archipelago, with the exception of Flores and Santa Maria, where the apiaries remained unaffected by either *Nosema* species [7].

The microsporidian *N. ceranae* exhibited the highest degree of prevalence among pathogenic agents in apiaries situated in Bulgaria. The absence of *N. apis*, which is indigenous to Europe, suggests that it has been eradicated from the aforementioned apiaries [8].

*Crithidia acanthocephali*, which was first identified in the digestive tract of Hemiptera, has the capacity to inhabit the gastrointestinal tract of honey bees. By means of artificial inoculation with this trypanosomatid, it has been observed that the flagella undergo a transformation, resulting in the formation of an adhesive pad that enables the parasite to fasten itself onto the gut wall by way of hemidesmosome-like junctions. Nevertheless, the effects of this species on bee welfare and its pathogenic mechanisms remain to be elucidated [9].

The occurrence of DWV was assessed in three commercial colonies of *Bombus terrestris*, and workers with wing deformities were detected. All tested specimens were found to be positive for replicative DWV, but adults showing deformities exhibited higher viral abundance compared to asymptomatic individuals. Additionally, viral infections were detected in the heads of these affected individuals. A sequence analysis of the DWV amplicons obtained indicated that they were characteristic of a strain that had been previously isolated in the United Kingdom [10].

A comparative investigation was conducted to evaluate the sensitivity and diagnostic accuracy of two field techniques for detecting *V. destructor* infestations in beehives, namely the sugar roll test and carbon dioxide (CO_2_) injection. The findings of the study revealed that the sugar roll technique was significantly more efficient and less hazardous compared to CO_2_ injection and afforded greater accuracy when diagnosing *V. destructor* infestations [11].

In vitro assays were carried out to examine the inhibitory activity of four strains of *Lactiplantibacillus plantarum* and four strains of *Apilactobacillus kunkeei*, which were isolated from the gastrointestinal tract of honey bees, against *Paenibacillus larvae* and *Melissococccus plutonius*. The outcomes of the study underscored the antimicrobial, biochemical, and cell surface attributes of these lactic acid bacteria, which are promising probiotics for beekeeping and as biocontrol agents against both foulbrood diseases [12].

An in vitro study was conducted to evaluate the contact toxicity, fumigation effectiveness, and repellent property of *Origanum heracleoticum* L. essential oil against *V. destructor*. The results of the study indicated the marked efficacy of the essential oil against the mite, suggesting a possible alternative for use in the control of varroosis [13].

Ten essential oils derived from indigenous Sardinian aromatic plants were subjected to in vitro testing for their efficacy against *Ascosphaera apis*, the causative agent of chalkbrood. The oils that exhibited the highest efficacy were *Thymus herba-barona*, *Thymus capitatus*, and *Cinnamomum zeylanicum*, which demonstrated minimum fungicidal concentration and minimum sporicidal concentration values ranging from 200 to 400 ppm. Future investigations conducted in apiaries will facilitate the evaluation of the impact of these essential oils on bees and their potential residues in hive products [14].

## Figures and Tables

**Figure 1:**
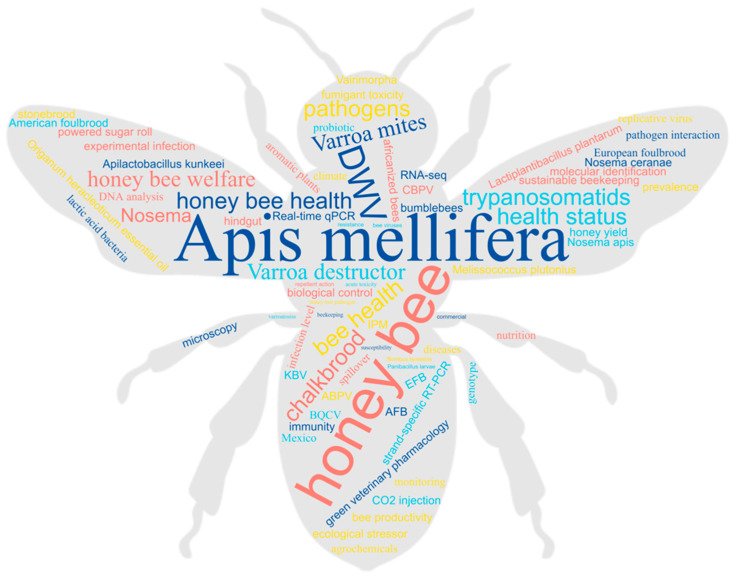
Word cloud generated using the keywords featured in each of the articles included in the Special Issue, with the size of each word corresponding to its frequency of appearance throughout the publications.
